# Adam Politzer (1835-1920): The Founder of Clinical Otology

**DOI:** 10.7759/cureus.68242

**Published:** 2024-08-30

**Authors:** Emily S Cushing, Taylor L Selby, Meredith Lehoe

**Affiliations:** 1 Medicine, Ohio University Heritage College of Osteopathic Medicine, Dublin, USA; 2 Otolaryngology - Head and Neck Surgery, Doctors Hospital-OhioHealth, Columbus, USA

**Keywords:** middle ear disease, middle ear ventilation, ear diseases, clinical otology, historical figures in medicine, historical vingette

## Abstract

Adam Politzer was a Hungarian surgeon and medical scientist, credited with describing the cochlear nucleus and otitis media, revolutionizing its treatment through his invention of “Politzerization.” After receiving training from notable medical figures in Vienna and London, where he studied and trained as a surgeon respectively, he became Vienna’s first professor of otology. In 1873, he established the first dedicated otology clinic in the world. His five-volume textbook, "Lehrbuch der Ohrenheilkunde,” unified otologic knowledge in his time and remains a resource to this day. Politzer’s contributions continue to influence modern otology, solidifying his legacy as a pioneering leader in medicine.

## Introduction and background

**Figure 1:**
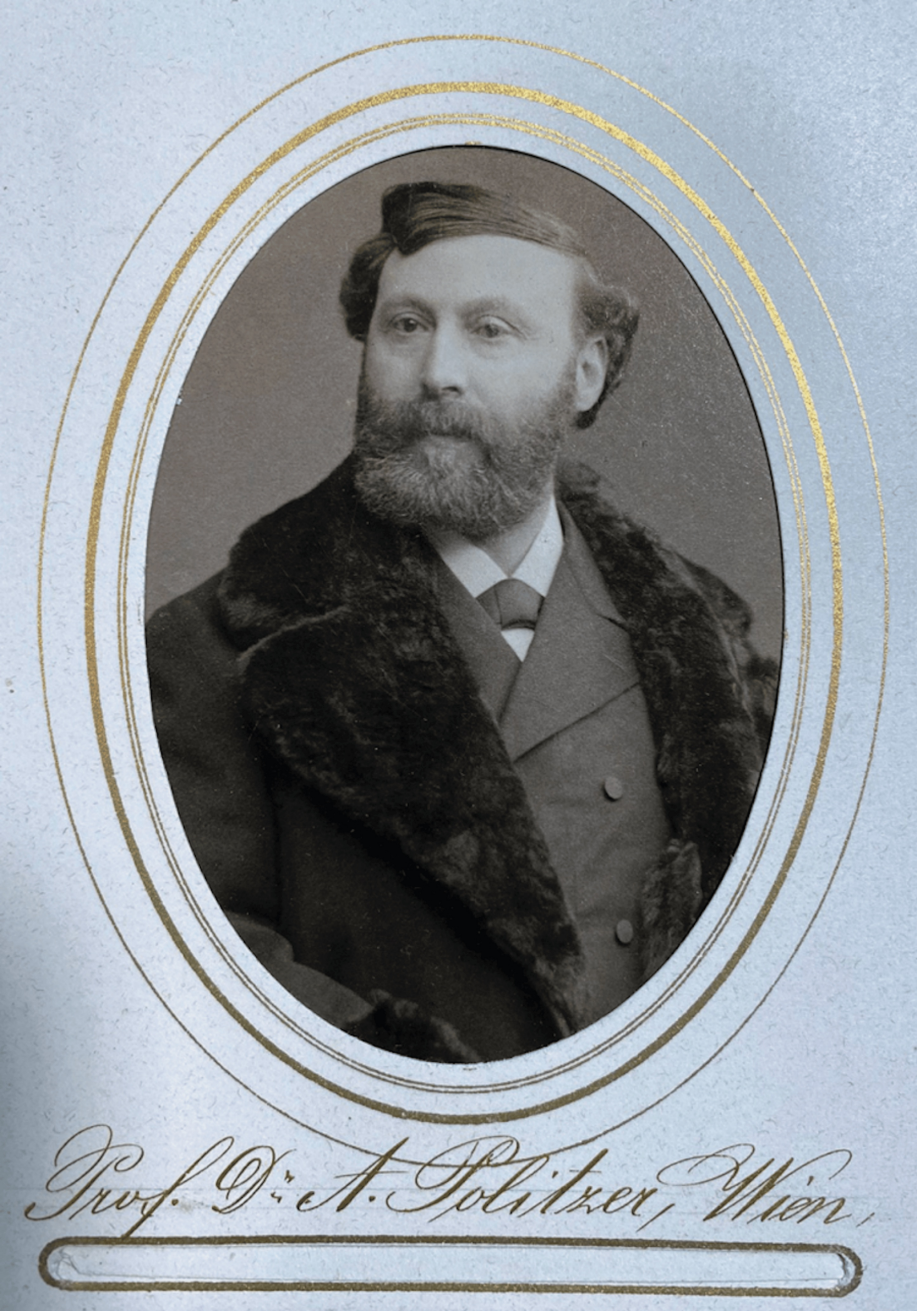
Adam Politzer, 1835-1920 Image courtesy: The Mudry Library [1].

The primary aim of this article is to emphasize the lasting legacy and contribution of Adam Politzer to the field of otology. Politzer was a surgeon and medical scientist (Figure 1). His early education in Kecskemet and Pest, Hungary, followed by medical training at the University of Vienna, formed the foundation for a notable career that would go on to reshape the understanding and treatment of ear diseases [1,2].

During Politzer’s time, diagnostic otology knowledge was limited. His passion for laboratory medicine and his detailed anatomical studies and prosecutions led to the first description of otitis media and the path of the cochlear nerve, aspects of otology that are of major importance to this day [2]. His trailblazing work, including the development of the “Politzerization” method and the establishment of the first otology clinic, paved the way for modern ear disease diagnosis and treatment [3]. Although the introduction of antibiotics, vaccines, and advances in microbiology have shifted practices away from some of Politzer's methods, his contributions undeniably laid the foundation for the current knowledge and treatment of otitis media [2,3]. It is because of this legacy that the International Society of Otology bears his name [2].

## Review

Politzer’s life and career

Adam Politzer was born on October 1, 1835, in Albertirsa, Hungary (Figure 1). He attended primary school in Kecskemet and completed his high school in Pest [1]. Afterward, he pursued medical studies at the University of Vienna in Austria, earning his medical degree in 1859 [2].

During his time in Vienna, Politzer was mentored by notable figures such as Kari Ludwig, Joseph Hyrtl, and Johann von Oppolzer [1]. His fascination with laboratory medicine emerged swiftly, leading him to focus on the innervation of the inner ear and the influence of pressure in the middle and tympanic cavities for his initial research [2]. Due to his detailed anatomical studies, he was the first to describe otitis media [3,4]. Later, he embarked to London to study under Josef Toynbee where he learned modern surgical techniques for the ear [2,4]. He returned to Vienna in 1861, becoming the sole professor of otology [2]. In 1873, he rose to prominence as the director and founder of the Clinic of Otology in Vienna, the first dedicated otology clinic of its time [5,6].

After completing his medical and surgical training, Politzer redirected his research endeavors. In 1876, he participated in the Centennial Exposition in Philadelphia, Pennsylvania. He displayed his collection of dissected temporal bones showing normal and diseased processes [4]. These dissections remain on display in museums in Vienna and Philadelphia nearly 150 years later [2,4]. He created many otologic medical devices such as the acoumeter, conical speculum, “hunting horn” hearing aid, and the handheld airbag for “politzerization,” for which he is most famous [2]. Politzer’s impact on the field of otology was profound. Despite retiring from active practice in 1906, he continued to contribute to the field through teaching and writing [4]. Adam Politzer died suddenly on September 8, 1920, in Vienna, Austria, leaving a lasting legacy that is still revered by many in the medical community today [1,2].

The cochlear nucleus

**Figure 2:**
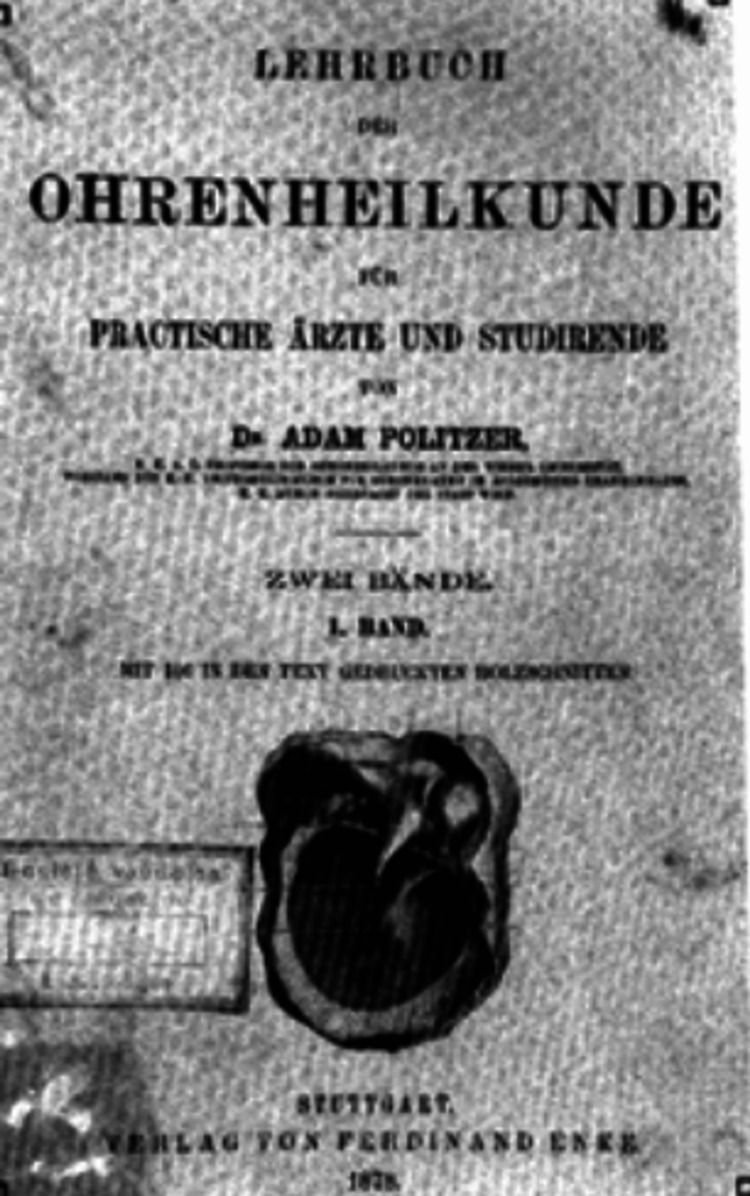
Lehrbuch der Ohrenheilkunde (Textbook of the Diseases of the Ear) Image courtesy: The Mudry Library [2].

Publications discussing the anatomical origin and course of the cochleovestibular nerve lacked detail and consistency during the nineteenth century [1]. Many pioneers of otology reported their findings and thoughts, but none integrated past knowledge into their discoveries. Politzer’s dedication to furthering the field of otology led to his five-volume textbook “Lehrbuch der Ohrenheilkunde” (Textbook of Disease of the Ear) (Figure 2). He was dedicated to continuously reviewing the literature to keep his textbook up to date for users. Before each new volume or edition, he would gather the latest discoveries and compile all relevant ideas, leading to a unification of concepts [7,8].

**Figure 3:**
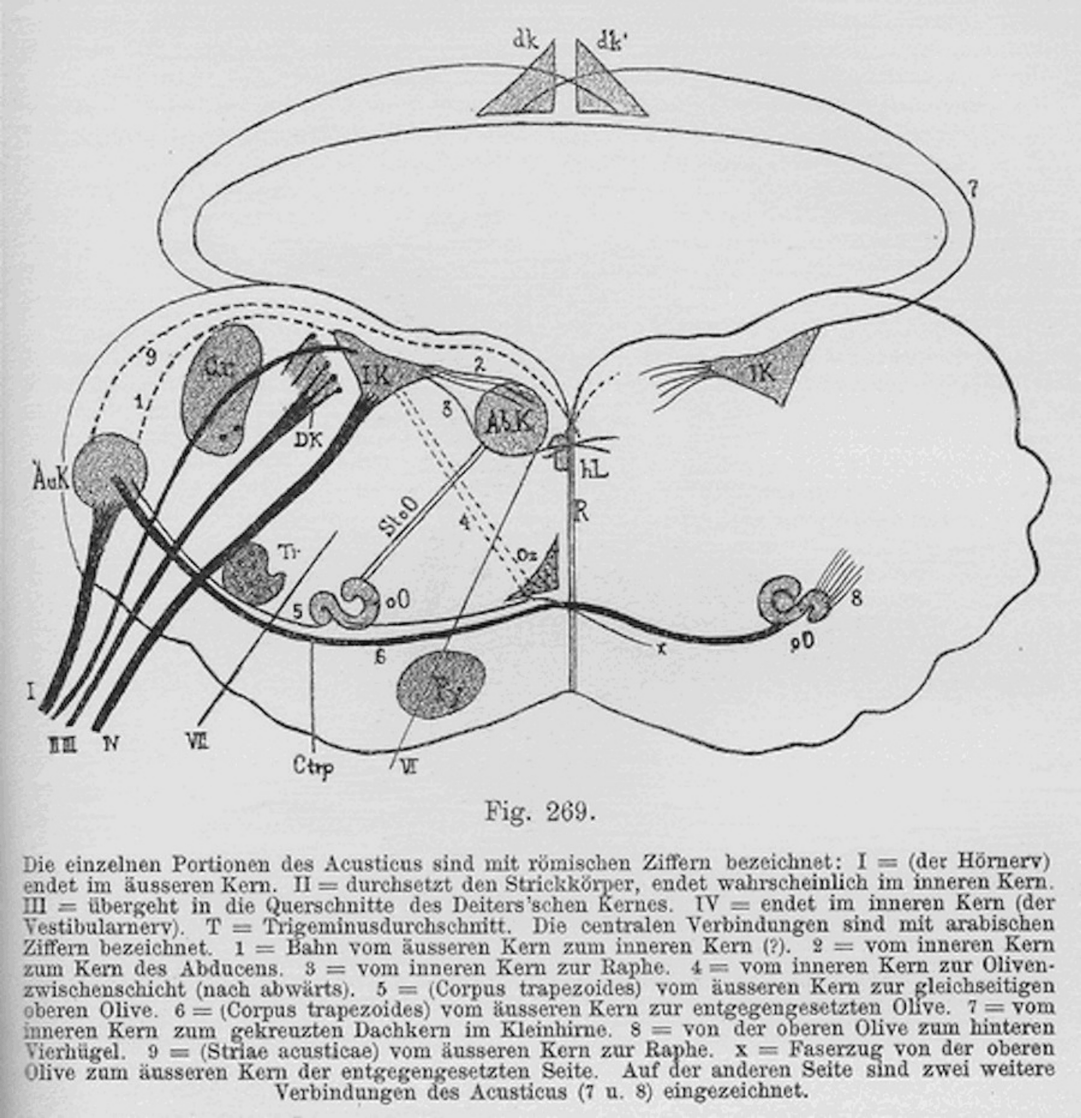
Politzer’s 1887 diagram of the auditory nucleus Image courtesy: The Mudry Library [1].

Through his research, Politzer made the connection that there was a bifurcation in the nerve course separating the cochlear and vestibular nerves [2]. He stated, “A part of the latter unites with fibers which arise in the ventral cochlear nucleus, run off in a lateral direction, and surround the corpus restiforme as the lateral acoustic nerve root” [1]. The discovery of the divergence led to more precise descriptions and depictions of both nerves (Figure 3). Integrating his neuroanatomical knowledge about the central auditory pathways allowed future generations to integrate the function of the auditory system with its structure.

Otitis media and the Politzer maneuver

**Figure 4:**
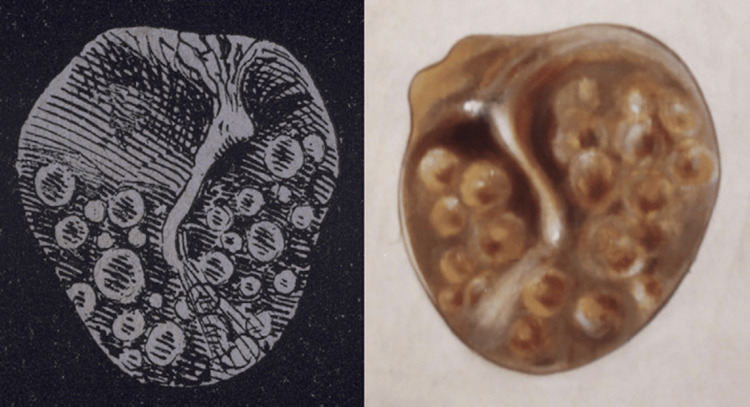
Politzer’s original drawing of mucus build-up of the middle ear, oil color painting Image courtesy: The Mudry Library [3].

The development of the conical ear speculum was revolutionary for the field of ontology, with Politzer even fashioning a design of his own [3]. The speculum unveiled an entirely new view of the ear, allowing physicians to view the middle ear cavity and tympanic membrane [3,5]. Currently, otitis media is regarded as a spectrum of diseases including otitis media with effusion, chronic suppurative otitis media, and acute otitis media [3]. Otitis media is diagnosed with the combination of physical examination (otoscopy), patient history, and presenting signs and symptoms. Various risks, including infections, environmental factors, and allergies, contribute to the development of otitis media [3]. A rudimentary definition of otitis media was first documented in Politzer’s writings in 1867 [3]. He describes “the presence of visible bubbles in the mucus … in the tympanic cavity. Very frequently I have seen them in children and adults who suffer from a bout of tonsillitis, severe cold or catarrh of the middle ear … of interest is the accumulation of serous fluid in the cavity, which has not been described, but which can be diagnosed by inspection” (Figure 4) [3]. These findings were illustrated, described, and published in his textbook, a textbook that lives on as a succinct account of modern otology knowledge [3]. In light of what is now known about the spectrum of otitis media, Politzer's description better aligns with otitis media with effusion, which involves the accumulation of serous fluid due to Eustachian tube dysfunction [3].

The impact of Politzer’s emphasis on direct otologic examination of patients cannot be overstated, as it remains a cornerstone of otology diagnosis to this day. Politzer directly witnessed the large number of people suffering from otitis media with effusion, a condition that remains prevalent today, and decided to find a way to treat it.

**Figure 5:**
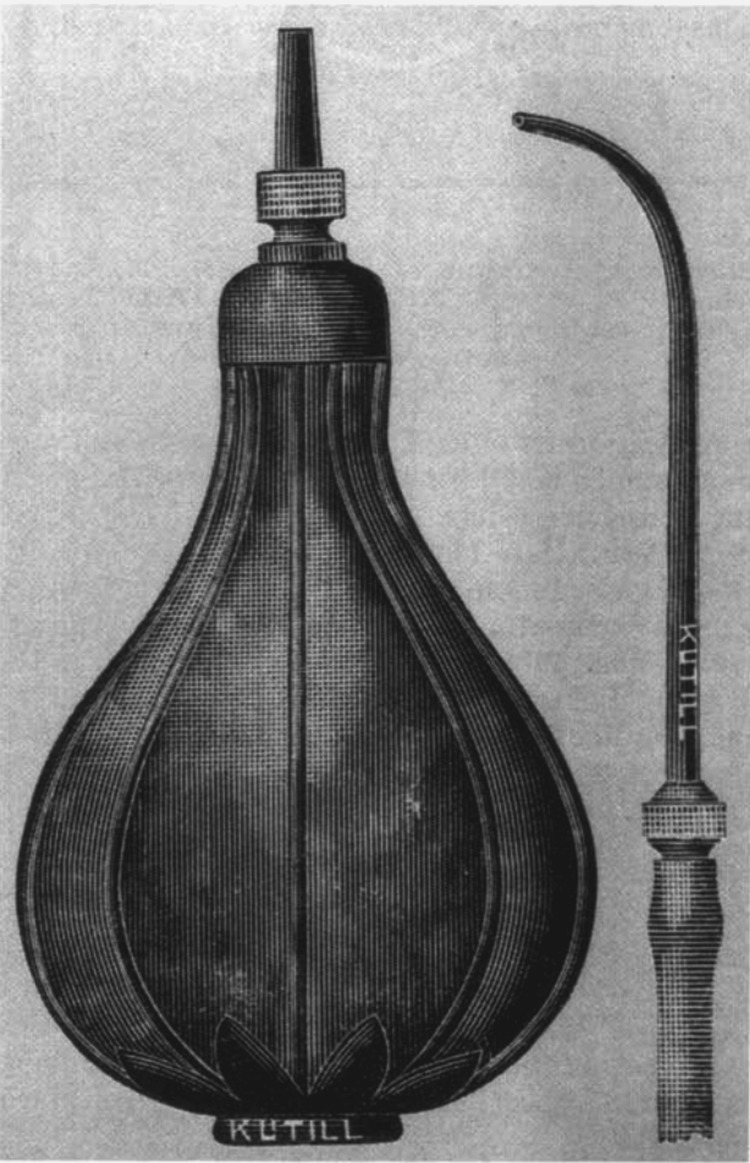
“Politzerization” method tool for eustachian tube dysfunction Image courtesy: The Weir Library [7].

In 1863, he described “Politzerization” in his work “My Method” [3,8]. The insufflation method refers to a “rubber bag inserted with a suitable nasal tip into the nasal vestibule and while the patient swallows a mouthful of water, the operator sharply squeezes the bag” (Figure 5). During the procedure, the operator uses an “auscultation tube,” one end goes into the patient's ear and the other into the operator’s ear [3,5]. While the bag is being squeezed, the operator can hear different auscultatory phenomena. Politzer describes “a patent eustachian tube has a blowing sound … an obstructed tube has less distinct blowing and if there are effusions present … faint cracklings are apparent” [3,5]. This device revolutionized the treatment of otitis media with effusion. Before “Politzerization,” sedated invasive eustachian catheterization was the only treatment to clear the effusion [5]. This technique only required a tube in the nose and the operator or otologist could hear the results in real-time [5]. However, no technique is without risk. Since this procedure requires patient cooperation, coordination between the operator and a well-educated, oriented patient is paramount [3,5]. Nonetheless, a variation of “Politzerization” is still practiced by many otologists today.

## Conclusions

Adam Politzer’s pioneering work in otology is monumental, laying the foundation for modern ear disease diagnosis and treatment, particularly with otitis media. Politzer’s commitment to innovation, education, and research led to the development of essential otologic techniques and tools, such as the Politzer maneuver. Politzer’s comprehensive textbooks have unified and advanced the field of otology, serving as invaluable resources for future generations. Adam Politzer’s legacy as a leader and pioneer in otology remains deeply respected, ensuring his place in the history of medicine.
